# Evaluation of the Rapid Scale-up of Collaborative TB/HIV Activities in TB Facilities in Rwanda, 2005-2009

**DOI:** 10.1186/1471-2458-11-550

**Published:** 2011-07-11

**Authors:** Eric S Pevzner, Greet Vandebriel, David W Lowrance, Michel Gasana, Alyssa Finlay

**Affiliations:** 1 Division of TB Elimination, U.S. Centers for Disease Control and Prevention, Atlanta, GA, USA; 2 International Center for AIDS Care and Treatment Programs, Columbia University, Kigali, Rwanda; 3 Global AIDS Program, U.S. Centers for Disease Control and Prevention, Kigali, Rwanda; 4 Rwanda Biomedical Centre, National TB Programme, Kigali, Rwanda

## Abstract

**Background:**

In 2005, Rwanda drafted a national TB/HIV policy and began scaling-up collaborative TB/HIV activities. Prior to the scale-up, we evaluated existing TB/HIV practices, possible barriers to policy and programmatic implementation, and patient treatment outcomes. We then used our evaluation data as a baseline for evaluating the national scale-up of collaborative TB/HIV activities from 2005 through 2009.

**Methods:**

Our baseline evaluation included a cross-sectional evaluation of 23/161 TB clinics. We conducted structured interviews with patients and clinic staff and reviewed TB registers and patient records to assess HIV testing practices, provision of HIV care and treatment for people with TB that tested positive for HIV, and patients' TB treatment outcomes. Following our baseline evaluation, we used nationally representative TB/HIV surveillance data to monitor the scale-up of collaborative TB/HIV activities

**Results:**

Of 207 patients interviewed, 76% were offered HIV testing, 99% accepted, and 49% reported positive test results. Of 40 staff interviewed, 68% reported offering HIV testing to >50% of patients. From 2005-2009, scaled-up TB/HIV activities resulted in increased HIV testing of patients with TB (69% to 97%) and provision of cotrimoxazole (15% to 92%) and antiretroviral therapy (13% to 49%) for patients with TB disease and HIV infection (TB/HIV). The risk of death among patients with TB/HIV relative to patients with TB not infected with HIV declined from 2005 (RR = 6.1, 95%CI 2.6, 14.0) to 2007 (RR = 1.8, 95%CI 1.68, 1.94).

**Conclusions:**

Our baseline evaluation highlighted that staff and patients were receptive to HIV testing. However, expanded access to testing, care, and treatment was needed based on the proportion of patients with TB having unknown HIV status and the high rate of HIV infection and poorer TB treatment outcomes for patients with TB/HIV. Following our evaluation, scale-up of TB/HIV services resulted in almost all patients with TB knowing their HIV status. Scale-up also resulted in dramatic increases in the uptake of lifesaving HIV care and treatment coinciding with a decline in the risk of death among patients with TB/HIV.

## Background

Tuberculosis (TB) is a treatable disease but remains the leading cause of death among persons living with HIV/AIDS (PLHIV) [1]. In some settings, up to 50% of patients with both TB disease and HIV infection die during TB treatment with most deaths occurring within 2 months of being diagnosed with TB [2-6]. In 2004, the World Health Organization (WHO) issued policy guidance on collaborative TB/HIV activities to reduce the burden of TB and HIV [7]. The interim policy recommended routine HIV testing for patients with TB disease and ensuring linkages to HIV care and treatment for patients with TB disease diagnosed with HIV infection (hereafter patients with both TB disease and HIV infection will be referred to as patients with TB/HIV). Also in 2004, WHO and UNAIDS issued a joint policy statement recommending routine provider-initiated HIV testing and counseling (PITC) for all patients with TB [8]. The policy recommendations were intended to increase the uptake of HIV testing among patients with TB by shifting from a model of people voluntarily seeking HIV counseling and testing (VCT) to providers initiating HIV testing and counseling (PITC). The shift from VCT to PITC resulted in TB clinics serving as points of entry for scale-up of HIV care and treatment services.

In 2005, Rwanda was experiencing a generalized HIV epidemic with 3% of the adult population living with HIV [9]. WHO estimated that the incidence of TB disease in Rwanda was 361 per 100,000 population and 41% of patients with TB disease were infected with HIV [10]. There were 161 TB clinics in Rwanda and all were using WHO's Directly Observed Treatment Short-Course (DOTS) strategy for the management of TB disease. New cases of pulmonary TB were treated with a standardized regimen of fixed-dose combinations of rifampicin, isoniazid, ethambutol, and pyrazinimide taken daily for two months (intensive phase) and then a fixed-dose combination tablet of rifampicin and isoniazid administered three days a week for four months (continuation phase). In 2005, patients with TB/HIV were eligible for antiretroviral therapy (ART) if they had a CD4^+ ^cell count < 350 cells/mm^3 ^[11]. For the majority of patients with TB/HIV, ART included efavirenz-based regimens initiated after completing the two month intensive phase of anti-TB treatment.

In response to the TB/HIV syndemic [12-14] and the new TB/HIV guidance from WHO, Rwanda's national TB program (NTP) drafted and approved a national policy on collaborative TB/HIV activities in December of 2005. The new national TB/HIV policy included provider-initiated HIV testing and counseling for all patients with TB as well as the provision of HIV care and treatment for patients with TB/HIV. Prior to implementing the policy and in the absence of nationally representative TB/HIV surveillance data, the NTP wanted to collect baseline data and identify possible barriers to routine HIV testing for patients with TB.

In October 2005, we evaluated current TB/HIV practices in health facilities providing TB services and identified possible barriers to implementing the new TB/HIV policy. The objectives of our evaluation were to 1) collect data to inform the national scale-up of HIV services in TB facilities, and to 2) provide baseline data by which to evaluate the scale-up of activities using national surveillance data. To inform the scale-up of activities we focused on assessing staff awareness of the relationship between TB and HIV, activities in support of TB/HIV coordination, determining what proportion of patients with TB were being offered HIV testing, evaluating the acceptability of HIV testing among patients with TB, and documenting whether or not patients with TB/HIV were receiving cotrimoxazole prophylaxis (CTX) and ART.

## Methods

### Baseline Evaluation

We conducted the evaluation in a non-probability sample of 23 of 161 (14%) TB clinics in Rwanda. The sample of TB clinics was selected by the NTP to represent all provinces of the country and include a mix of urban and rural health centers and hospitals providing TB diagnostic and treatment services.

The three evaluation components were 1) structured interviews with patients arriving at the clinics to receive their anti-TB medications, 2) structured interviews with staff working at the TB clinics, and 3) review of the TB registers and patient treatment cards for patients registered for anti-TB treatment during a specified three month period. All data collection forms were piloted and revised based on focus group discussions with patients receiving anti-TB treatment and staff working at the TB clinics of two sites not selected to participate in the evaluation. Data collectors fluent in Kinyarwanda, French, and English were trained to lead the focus group discussions. Data collection forms, focus group guides, and resultant transcripts were translated from English to Kinyarwanda and then back translated to ensure proper translation.

From October 1 to October 14, 2005, we collected baseline data using two teams consisting of staff from the NTP, CDC, and medical students from the National University of Rwanda. All data collectors participated in a two-day skills-based training on leading focus groups, conducting structured interviews, using the data collection tools, data entry, and ensuring the protection of study participants.

#### Structured interviews with patients

We approached the first 10 patients arriving for anti-TB treatment at each clinic. After receiving oral informed consent, we conducted structured interviews including both open and closed-ended questions. We interviewed patients to assess their experiences with HIV testing with a focus on what encouraged them or prevented them from getting HIV testing. For patients who disclosed being infected with HIV, we asked whether or not they were receiving CTX and ART. For patients not tested, we asked if they would accept HIV testing if offered and, if not, why?

#### Structured interviews with staff

We interviewed 1-2 staff at each clinic, depending on the number of staff working at the clinic and their work burden at the time of our visit. We asked staff open and closed-ended questions to assess their awareness of the association between TB and HIV, their knowledge of risk factors associated with TB disease, and the percentage of patients with TB offered HIV testing and counseling. We also asked them to describe any perceived barriers to offering HIV testing to patients with TB. Lastly, we asked about services routinely provided for patients with TB/HIV.

#### Review of TB registers and patient treatment cards

We reviewed the TB registers and patient treatment cards for all patients registered for anti-TB treatment during the fourth quarter of 2004. We selected the fourth quarter of 2004, because we wanted to evaluate how HIV impacted the risk of death among people with TB and at the time of our evaluation these patients were the most recent quarterly cohort with documented treatment outcomes. Registers were reviewed to determine the proportion of patients with documented HIV test results prior to transitioning from VCT to PITC and to compare TB treatment outcomes for patients based on their HIV status. We abstracted data on patient demographics, TB diagnosis (pulmonary versus extrapulmonary), treatment category (new versus re-treatment), treatment outcome (cured, completed, died, defaulted, or transferred out), HIV status, and receipt of CTX and/or initiation of ART for patients with TB/HIV.

### Evaluating the Scale-up of Collaborative TB/HIV Activities

We measured the scale-up of collaborative TB/HIV activities by comparing our baseline evaluation data to select TB/HIV indicators routinely collected by all TB clinics in Rwanda and reported to the national TB program. District level reports are compiled and transmitted to the NTP following quarterly district-level evaluation meetings. All districts report to the NTP and the quality and completeness of the TB/HIV data are routinely evaluated during supportive supervision visits. Using the nationally representative data provided by the Ministry of Health, we compared the proportion of patients with TB getting an HIV test, testing positive, and among those testing positive the proportion getting CTX and ART from 2005 to 2009. Our crude analysis of the risk of death among patients with TB/HIV relative to patients with TB not infected with HIV was limited to the years for which patients' anti-TB treatment outcomes could be linked to their HIV testing data (i.e., our baseline data and enhanced surveillance done in 2007).

### Statistical analysis

We calculated descriptive statistics for data from each of the three components of the evaluation. We performed Pearson's Χ^2 ^test to examine the association between dying during anti-TB treatment and patients' HIV status. For our crude analysis of the risk of death during anti-TB treatment, we excluded patients who defaulted or transferred out because we could not determine whether they survived or died.

### Ethical approval

The evaluation protocol underwent ethical review and was determined to be a program evaluation and not research by CDC, the Rwandan Ministry of Health, and Columbia University.

## Results

### Baseline Evaluation

All geographic regions of the country were represented by the sample of 23 TB clinics and the geographic distribution of the sample approximated the distribution of clinics nationally (Table 1). Our sample underrepresented health centers (48% of our sample relative to 70% nationally) and overrepresented district hospitals (44% of our sample relative to 20% nationally). On-site HIV testing and ART services were available at 74% and 65% of the health facilities in our sample compared to 70% and 41% of facilities nationally.

**Table 1:** Characteristics of evaluation sites and all TB facilities in Rwanda, 2005

	Evaluation Sites (n = 23)		All TB Facilities (N = 161)	
	
	**No**.	%	**No**.	%
Province				
Kigali City	4	18	22	14
Northern	5	22	22	14
Southern	6	26	42	26
Eastern	4	17	35	22
Western	4	17	40	24
Facility type				
Health center	11	48	113	70
District hospital	10	44	33	20
Referral hospital	2	8	4	3
Other	0	0	11	7
On-site HIV services				
VCT*	17	74	113	70
ART^†^	15	65	66	41

* VCT = Voluntary HIV Counseling and Testing

^†^ARV = antiretroviral therapy

#### Structured interviews with patients

Of the 207 patients with TB we interviewed, 81 (39%) were female, median age was 37 years, 113 (55%) had completed primary school and 63 (30%) had no formal education. We interviewed an average of nine patients per site depending on the number of patients presenting at the clinics for treatment at the time of our site visits (range 1 to 14 patients interviewed per site). Patient consent to participate was nearly 100% with refusals being so rare that data collectors stopped routinely documenting them.

**Figure 1:**
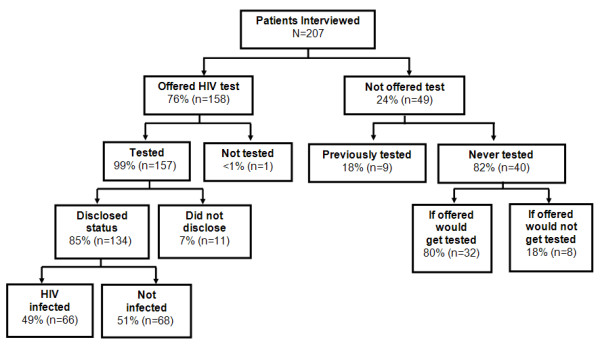
**HIV Testing Among Patients with TB**.

Of the 207 patients interviewed, 158 (76%) reported being offered an HIV test at the time of TB diagnosis. Of those, 157 (99%) accepted testing and received their results (Figure 1). Among 134 (85%) patients who disclosed their test results to interviewers, 66 (49%) reported a positive HIV test result. Among the 49 patients not offered a test, 9 had been previously tested and 32 of the 40 not previously tested (80%) responded that they would accept HIV testing if it were offered to them by staff at the TB clinic. Overall, 81% (167/207) of patients knew their status either because they were offered and accepted testing or they had been previously tested.

Patients who reported accepting HIV testing in TB services (n = 157) were asked what convinced them to get tested. Patients could provide multiple responses with the most common reasons cited for getting an HIV test being the patient's desire to know their HIV status (71%, n = 110), recommendation by a health care worker (35%, n = 55), and possible exposure to HIV through unprotected sexual intercourse (25%, n = 38). Among the seven patients not offered a test, not previously tested, and who would refuse a test if offered; the most common reasons cited for not getting tested were that they did not perceive themselves to be at risk for HIV infection (n = 4) or feared a positive test result (n = 2).

Patients with TB/HIV were asked whether someone talked with them about starting CTX and/or ART and whether they had initiated ART. For the 66 patients who disclosed a positive HIV test result, 97% (64/66) recalled talking with someone about taking CTX to prevent opportunistic infections, 89% (59/66) reported meeting with someone to discuss ART, and 53% (35/66) responded that they were currently receiving ART.

#### Structured interviews with staff

We interviewed 40 staff from the 23 TB clinics, including 35 (88%) nurses, one nurse assistant, one medical assistant, one TB/HIV focal person, and one health center director. Staff awareness of the relationship between TB and HIV was evaluated by asking open-ended questions about risk factors for developing TB disease and the leading cause of death among PLHIV. Among the 40 staff, 35% (14/40) correctly identified TB disease as the leading cause of death among PLHIV. When asked to identify the most common risk factors for PLHIV developing TB disease, staff mentioned HIV infection or immune suppression (83%), contact with another person with TB disease (75%), malnutrition (58%), and poverty (13%).

When asked about the percentage of patients with TB offered HIV testing at their TB clinics, 11 (32%) staff reported their clinic offered testing to all patients, 16 (47%) reported their clinic offered to more than half of patients, seven (21%) to less than half of patients, and six did not know. We then asked staff to talk about barriers to offering HIV testing to patients with TB. The most frequently mentioned barriers as perceived by staff were lack of trained staff (43%), insufficient space for testing and counseling (33%), and patient concerns about stigmatization associated with HIV testing (25%) (Table 2).

**Table 2:** Barriers to offering HIV testing reported by TB clinic staff

	n*	(%)
Not enough trained staff	17	(43)
Not enough space	13	(33)
Patient's concern about stigma of test	10	(25)
Responsibility of VCT clinic	6	(15)
Not enough supplies	5	(13)
Staff uncomfortable offering the test	4	(10)

* Staff could give multiple responses

Lastly, we asked staff about services routinely provided for patients with TB after HIV testing and counseling. For patients with a positive HIV test result, 90% (36/40) of staff reported that patients at their facility receive counseling, 65% (26/40) receive ART, 65% (26/40) CTX, 65% condoms, and 10% (4/40) nutritional support. For patients with a negative HIV test result, 93% (37/40) of staff reported providing counseling on risk reduction, 58% (23/40) assessed patient's risk of HIV infection, 35% (14/40) offered condoms only to patients believed to be at high risk for HIV infection and 10% (4/40) offered condoms to all patients.

#### Review of TB registers and patient treatment cards

During the fourth quarter of 2004 (the most recent cohort with treatment outcomes at the time of our baseline evaluation) there were 542 patients registered for anti-TB treatment at the 23 TB clinics; 211 (39%) were female and the mean age of patients was 32.8 years (SD 14.1) (Table 3). Overall, 77% (n = 416) of patients were diagnosed with pulmonary disease and 80% (435/542) were new patients with no known history of being previously treated for TB disease. HIV test results were documented in the TB register for 48% of patients (n = 258), in the patient treatment cards for 38% (n = 208), and either the TB register or patient treatment cards for 52% (n = 282) of patients. Among patients with a documented HIV test, 44% (122/277) had a positive test result. Of the 122 patients with a positive HIV test result, there was documentation in the register or their patient treatment cards that 2.5% (3/122) were receiving CTX and 12.3% (15/122) had initiated ART.

**Table 3:** Patient and disease characteristics and TB treatment outcomes (N = 542 patients registered during the 4^th ^Quarter of 2004)

Characteristic	**No**.	(%)
Female	211	(38.9)
Mean age in years	32.8	SD*14.2
Pulmonary TB	416	(76.8)
New patient	435	(80.3)
HIV Status		
Positive	122	(22.5)
Negative	155	(28.6)
Not documented	265	(48.9)
Treatment outcomes^†^		
Cured	198	(36.5)
Completed	138	(25.5)
Died	60	(11.1)
Defaulted	15	(2.8)
Transferred out	76	(14.0)

*SD = standard deviation

^† ^Missing outcomes for 10.1% (n = 55) of patients registered

Among patients with documented anti-TB treatment outcomes, 69% (n = 336) had successful outcomes (i.e., either cured or completed) and 11.1% (n = 60) died before completing anti-TB treatment. Treatment outcomes differed based on patients' HIV status (Table 4). The crude risk of death among patients with TB/HIV was 6.1 (95% CI 2.6, 14.0) times the risk among patients with TB not infected with HIV. The crude risk of death among patients with TB and undocumented HIV status was 2.9 (95% CI 1.2, 6.9) times the risk among patients with TB not infected with HIV.

**Table 4:** TB treatment outcomes* by HIV status for patients registered during the 4^th ^quarter of 2004 (n = 358)

	Treatment success		Died		Risk of death	
	
	n	(%)	N	(%)	RR	95% CI
HIV positive (n = 90)	63	(70)	27	(30)	6.1	(2.6 -14.0)
HIV negative (n = 121)	115	(95)	6	(5)	Referent	Referent
HIV unknown (n = 147)	126	(86)	21	(14)	2.9	(1.2-6.9)

* Analysis limited to patients with anti-TB treatment outcomes of success (cured and completed) or death

### Evaluation of the Scale-up of Collaborative TB/HIV Activities

**Figure 2:**
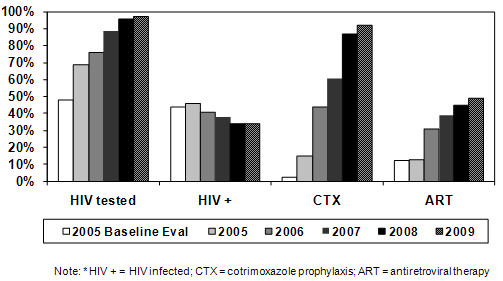
**Trends in HIV testing, HIV infection, and HIV care and treatment for patients with TB in Rwanda, baseline evaluation data from 2005 and nationally reported data from 2005 - 2009**.

The proportion of patients with TB having HIV test results documented in the TB register increased from 48% during our baseline evaluation to 69% during the first year of scale-up in 2005, and then increased to 97% in 2009 (Figure 2). Provision of CTX for people with TB/HIV increased from the 2.5% noted during our evaluation to 15% in 2005 and then to 92% in 2009 [12,13]. The proportion of people with TB/HIV initiating ART increased from the 12.5% at baseline to 13% in 2005 and 49% in 2009 [15,16]. The most recent year with reported data on anti-TB treatment outcomes stratified by HIV status was 2007 and the risk of death during anti-TB treatment among patients with TB/HIV was 1.8 times greater (95% CI 1.68, 1.94) than the risk among patients with TB not infected with HIV [16]. The relative risk of death in 2007 (RR = 1.8, 95% CI 1.68, 1.94) was less than the relative risk calculated for patients in our 2005 baseline evaluation with confidence intervals that did not overlap (RR = 6.1, 95% CI 2.6, 14.0).

## Discussion

We conducted this evaluation to collect baseline data to support and inform the implementation and ongoing scale-up of the new national TB/HIV policy in Rwanda. Multiple data sources were used in order to compare and contrast what patients reported, what staff reported, and what was documented about HIV testing for patients with TB and care and treatment for patients with TB/HIV. Our interpretations of the evaluation data were that patients with TB and staff at TB clinics were receptive to HIV testing which supports the premise that TB clinics could be an important point of entry into life-saving HIV care and treatment services in Rwanda. Review of subsequent programmatic data confirms the success of the national scale-up of key TB/HIV services and suggests a substantial public health impact from these efforts.

From patient interviews we learned that when offered, almost all patients (99%) reported accepting HIV testing. The almost universal acceptance of HIV testing by patients and the stated willingness to accept testing by patients not offered HIV testing, suggested that implementing routine PITC for all patients with TB disease could dramatically increase testing uptake. However, interviews with TB clinic staff revealed that less than a third of staff reported offering HIV testing to all patients with TB and only 38% knew that TB was the leading cause of death among PLHIV. Additionally, our review of patient records revealed that there was no documentation of HIV test results for almost half (48%) of patients registered for TB treatment. The lack of documentation indicated that staff were either not routinely offering or documenting HIV testing. Inconsistent documentation of HIV information could result in missed opportunities for eligible patients to initiate ART and underestimation of the burden of disease and resources needed to treat TB/HIV. Similarly, based on available data it was not possible to determine how much of the change in the proportion of patients receiving CTX or ART is due to enhanced services versus improved data recording and reporting. These discrepancies identified during our evaluation highlight the importance of routinely evaluating surveillance systems to ensure completeness and accuracy of surveillance data. To support the scale-up of TB/HIV activities, resources were needed to train staff about the relationship between TB/HIV and the importance of routinely offering HIV testing and documenting patients' test results.

The proportion of patients with TB disease and HIV infection based on patient interviews (49%) and clinical record reviews (42%) were consistent with the national data (46%) in 2005. Patients with TB and HIV infection were more likely to die during anti-TB treatment than patients with TB not infected with HIV. Also, our finding that patients with unknown HIV status had a greater risk of death than patients with HIV negative test results suggests that the group with unknown HIV serostatus probably contained people with undiagnosed HIV. Our finding of an increased risk of death during anti-TB treatment for people infected with HIV is consistent with other published reports [17,18] and highlights the importance of routine HIV testing to minimize delays in HIV diagnosis and initiation of ART to reduce the risk of death among patients with TB/HIV [19-21]. Expanded access to HIV testing, care, and treatment were urgently needed based on the proportion of patients with unknown HIV status, the high rate of HIV infection coupled with poorer TB treatment outcomes, and the lack of documented CTX and ART for patients with TB/HIV.

In response to the evaluation findings, the MOH modified their draft TB/HIV policy in November 2005, to specify that CTX should be routinely provided through the TB program for all patients with TB/HIV. Evaluation findings were used to plan and implement national, regional, and district trainings on PITC and to ensure that patients with TB/HIV receive CTX and ART when indicated. The National TB and HIV Programs led the implementation of TB/HIV collaborative activities, starting with revision of program guidelines, training materials, and monitoring and evaluation tools. TB/HIV collaborative activities were implemented and refined at two model centers prior to national scale-up. An assessment was conducted to identify the training needs of health service staff to inform the development and decentralized implementation of an integrated TB/HIV curriculum. The curriculum emphasized cross-training and was implemented with staff at district-level health facilities providing quality assured TB diagnostic and treatment services and ART. The two model centers were used as practical training centers and implementation fidelity was assured through intensive site support visits that included clinical mentorship and ongoing monitoring and evaluation of key indicators.

Rwanda has achieved dramatic increases in HIV testing and provision of CTX and ART for patients with TB/HIV [22-24]. The risk of death among patients with TB/HIV has decreased as HIV testing and the provision of CTX and ART has been scaled-up. The noted decrease in the relative risk of death among patients with TB/HIV (from 6.1 to 1.8 with non-overlapping confidence intervals) is encouraging but should be interpreted with caution because the comparison is based on cross-sectional data.

In 2006, in response to increasing evidence that early initiation of ART substantially improves the survival of patients with TB/HIV [25], Rwanda began implementing a "one-stop" TB-HIV integrated services model where ART is provided to patients with TB/HIV at TB Diagnostic and Treatment Centers (DTC). By April 2009, the "one-stop" TB-HIV integration model had already been adopted by 80% (153 of 192) of TB clinics (personal communication with Director of the NTP), which should further expand ART coverage for people with TB/HIV. The NTP aims to reach 100% coverage of CTX as it is a relatively inexpensive, safe, and life-saving intervention [26]. Evaluation research is now needed to document the operationalization and impact of the "one-stop" approach, especially in light of the WHO's "3 I's" initiative (intensified TB case-finding, isoniazid preventive therapy, and infection control), which calls for greater integration and provision of services for people with TB/HIV [27].

Our evaluation findings were based on a non-probability sample of TB clinics, staff, and patients, and therefore may not have been representative of all TB clinics in the country. Despite the higher proportions of district and referral hospital-based clinics in our non-probability sample compared with national TB clinics, we do not believe that the former was biased towards selecting "top-performing sites," as the performance indicators (e.g., HIV testing, provision of CTX/ART) from our sample were lower than national estimates from 2005. Data from patients and health care workers were based on self-report and therefore are subject to recall and social desirability bias. Data from healthcare workers or TB registers on the provision of ART underestimate the uptake of ART because the denominator includes an undeterminable number of patients with TB who were not eligible for ART based on their CD4 cell counts (note: the CD4 threshold below which people are eligible for ARVs increased from 200 to 350 in 2008). We were unable to ascertain the true outcome status of patients receiving anti-TB treatment who had defaulted, which may have resulted in an underestimation of mortality. Despite the aforementioned limitations, our baseline estimates of HIV infection among patients with TB and provision of CTX and ART for patients with TB/HIV were consistent with 2005 national estimates that were released after our evaluation. We were not able to perform survival analyses and our estimates of the risk of death during TB treatment are unadjusted because patient-level data for possible confounders were not available.

## Conclusions

Using three sources of data, we were able to demonstrate that patients and staff were receptive to HIV testing and that TB clinics could serve as a point of entry into HIV care and treatment. Patients' acceptance of and staff willingness to offer HIV testing refuted concerns that stigma or increased work load were barriers to routine PITC and provided further evidence for routinely offering testing. Introduction of PITC during rapid scale-up of HIV care and treatment services in Rwanda resulted in people being diagnosed and treated for HIV who otherwise would likely have had delayed diagnoses or died of undiagnosed HIV disease [28,29]. Rwanda provides a model for integrating TB-HIV services that has resulted in the majority of patients with TB knowing their HIV status and a dramatic scale-up of lifesaving HIV care and treatment that coincided with a decline in the risk of death among patients with TB/HIV.

## Competing interests

The authors declare that they have no competing interests.

## Authors' contributions

EP has full access to all the study data and accepts responsibility for the integrity and accuracy of the data and data analysis. EP, GV, MG, and AF conceptualized and designed the study. Acquisition of data was done by EP, GV, and AF Statistical analyses were done by EP, DL, AF, and interpretation of the data was done by EP, GV, DL, MG, and AF Drafting of the manuscript was done by EP and DL with critical revisions for intellectual content provided by GV and AF. All authors have read and approved the final manuscript.

## Pre-publication history

The pre-publication history for this paper can be accessed here:

http://www.biomedcentral.com/1471-2458/11/550/prepub
